# Electrochemical Evaluation of the Compact and Nanotubular Oxide Layer Destruction under Ex Vivo Ti6Al4V ELI Transpedicular Screw Implantation

**DOI:** 10.3390/ma13010176

**Published:** 2020-01-01

**Authors:** Katarzyna Arkusz, Marta Nycz, Ewa Paradowska

**Affiliations:** Department of Biomedical Engineering, Faculty of Mechanical Engineering, University of Zielona Gora, Licealna 9 Street, 65-417 Zielona Gora, Poland; m.nycz@ibem.uz.zgora.pl (M.N.); e.paradowska@ibem.uz.zgora.pl (E.P.)

**Keywords:** transpedicular screw, titania nanotubes, ex vivo implantation, microrupture

## Abstract

Nano-engineered implants are a promising orthopedic implant modification enhancing bioactivity and integration. Despite the lack of destruction of an oxide layer confirmed in ex vivo and in vivo implantation, the testing of a microrupture of an anodic layer initiating immune-inflammatory reaction is still underexplored. The aim of this work was to form the compact and nanotubular oxide layer on the Ti6Al4V ELI transpedicular screws and electrochemical detection of layer microrupture after implantation ex vivo by the Magerl technique using scanning electron microscopy and highly sensitive electrochemical methods. For the first time, the obtained results showed the ability to form the homogenous nanotubular layer on an Ti6Al4V ELI screw, both in α and β-phases, with favorable morphology, i.e., 35 ÷ 50 ± 5 nm diameter, 1500 ± 100 nm height. In contrast to previous studies, microrupture and degradation of both form layers were observed using ultrasensitive electrochemical methods. Mechanical stability and corrosion protection of nanotubular layer were significantly better when compared to compact oxide layer and bare Ti6Al4V ELI.

## 1. Introduction

The Ti6Al4V ELI (Grade 23) alloy is the most commonly used biomaterial for dental and orthopedic implants, and its advantages include good mechanical properties, machinability, biocompatibility, and excellent fatigue resistance [1]. However, the major concern of using this alloy in clinics is the presence of infiltrated aluminum and vanadium ions in its chemistry, which can potentially increase the expressions of pro-inflammatory factors and cause osteolysis, exhibiting a toxic effect in the body. Products of implant degradation in the form of metallic ions or corrosion products could influence intercellular space or penetrate cells, which leads to metallosis [2]. Therefore, surface treatment methods are very important in forming physicochemical protection and biocompatibility of titanium alloys.

To improve in vivo osteointegration, the Ti6Al4V ELI is subjected to surface treatments, such as nitriding, electropolishing, and electrochemical oxidation. Thermal oxidation [3] or plasma [4] is distinguished on the alloy surface from the processes of oxide layer production, but, more often, the anodic surface is subjected to anodizing [5,6,7,8]. The major advantages of the anodizing process are ability to control the fine-tuning of oxide film thickness, feature size, topography, and chemistry, as well as its simplicity, low-cost, ease of implementation, and scalability at the industrial level [6].

The electrochemical formation of compact and porous oxide layers on the surface of the Ti6Al4V alloy is difficult. The presence of vanadium favors the adsorption of anions, and, in addition, it is well dissolved in solutions containing fluoride and chloride ions [9]. The thickness of the film, the size, and distribution of the pores depend on the underlying substrate structure: the dissolution is slightly faster on top of the vanadium-enriched β-phase than on top of the α-phase. The thickness and topography of the oxide layers formed on the alloy depend, as in the case of titanium, on factors such as the electrolyte composition, current parameters, and anodizing time. The oxide layer controls the biological response by accelerating the formation of hydroxyapatite or osteoblastics in growth, and also reduces the formation of corrosive foci—the main sources of release of toxic metals into the bloodstream and surrounding tissues. In literature, nanostructures in the form of compact oxide films, disordered porous films, and self-organized porous and nanotubular films were fabricated by anodization using various electrolytes, such as NH_4_F, CH_3_COOH, H_2_SO_4_, HF, Na_2_HPO_4_, NaF, NaOH, and NH_4_Cl [10].

Macak et al. [11] and Filova et al. [12] anodized Ti6Al4V in 1 M (NH_4_)_2_SO_4_ electrolytes containing varying concentrations of NH_4_F and successfully obtained self-assembled nanotubular oxide films only in the α-phase. For the β-phase, enhanced etching was observed, leading to selective dissolution and inhomogeneous pore formation. Kulkarni et al. [13] examined the morphology of nanotubular oxide films in the α and β-phases of Ti6Al4V anodized in 1 M H_3_PO_4_ and 0.2 wt.% HF electrolyte. Similarly, only the α-phase exhibited nanotubular structures upon anodizing, while the β-phase did not form any nanostructures, indicative of different reactivities of each phase for fluoride containing electrolytes. Anodic oxidation in non-organic electrolyte proceeds with complete oxidation and formation of a nanotubular layer in the α-phase of Ti6Al4V and/or loss of the β-phase grains and formation of the nanotubes on the underlying α-phase matrix.

Park et al. [14] and Acquesta et al. [15] examined the growth behavior of nanotubular oxide on Ti6Al4V foil in glycerol solution containing NH_4_F. Anodized surfaces showed a wide distribution of nanotubular diameters and smooth side walls; nanotubes with smaller diameters formed across spaces were present between nanotubes with larger diameters. Interestingly, diameters of the nanotubular features increased toward the base of the nanotubes as opposed to the commonly observed tapered nanotubular morphology. Attempts to fabricate a nanotubular layer on the Ti6Al4V alloy by anodizing in ethylene glycol solution were performed by Saharudin et al. [16] and Fraoucene et al. [17]. With an increase in water content, the nanotubular structure is formed in the α + β phases with different morphological parameters. However, an inner diameter of nanotubes formed in α + β phases was between 81 and to 206 nm, which is not desirable for protein/cell adsorption and thus does not favor osteointegration [18]. Previous attempts of nanotubular layer formation on Ti6Al4V ELI foil [9] and on Ti6Al4V ELI cervical screws [19] allowed for the production of a layer of regular nanotubes with diameters ranging from 35–65 nm; these were rich in vanadium oxides in both (α + β) phases of the Ti6Al4V alloy by anodizing in 99.0% ethylene glycol with the addition of 0.5–0.7 wt.%. NH_4_F, but this layer was nonhomogeneous and cracked.

Regardless of the tremendous progress made in nanotubular surface modified implants, there are still numerous research gaps that prevent their successful shift to clinical testing and the commercial market. In fact, mechanical stress and abrasion can lead to the formation of cracks and delamination, and finally to complete implant failure.

Hitherto, several attempts have been made to produce the nanotubular oxide layer on orthopedic devices, such as screws, plates, etc. During clinical implantation, implants are subjected to high mechanical stresses, causing the degradation of the nanotubular layer and/or corrosion. The titania nanotubes (TNT) were successfully produced on Ti6Al7Nb screws [20], Ti CP2 screws [21], and Ti6Al4V ELI screws [19,22], etching to the Ti6Al4V screw [23]. Though the implantation process was not considered in this attempt, the formed nanotubular layer of TiO_2_ was cracked [19] or produced exclusively in the α-phase [20,21,22,23]. This is extremely urgent for obtaining the homogenous layer of compact and nanotubular oxide on the surface of implantable devices and to ensure protection against microrupture, ion release, and corrosion that has not yet been done.

The aim of this work was to produce a homogenous nanotubular and compact oxide layer on the Ti6Al4V ELI transpedicular screws, both in the α and β-phases and examine its delamination and chemical stability after ex vivo implantation with the use of the Magerl technique. The primary objective of this work is to evaluate how the TiO_2_ nanotubes degrade during implantation, and if TNT debris could influence tissue response.

Secondly, in the study, researchers examined corrosion resistance of a compact and nanotubular oxide layer before and after implantation. This examination allowed for determination of corrosion resistance of the implant in the state after implantation, without the additional procedure of screwing the implant, which has been analyzed so far. Finally, the measurement carried out helped researchers in the study prove that nanotubular layer modification makes clinical sense.

## 2. Materials and Methods

Transpedicular screws (5 mm in diameter and 40 mm in length, LfC, Zielona Gora, Poland) made of Ti6Al4V ELI purchased from DERO-SGL System (Self Guided Lock System) were used to correct and stabilize the thoracolumbar spine. Ethylene glycol (EG, purity 99.8%), ammonium fluoride (NH_4_F, purity ≥ 99.99%), sodium chloride (NaCl, purity ≥ 99.5%), phosphate-buffered saline (PBS, yields 0.01 M phosphate buffer, 0.0027 M potassium chloride, and 0.137 M sodium chloride, pH 7.4), acetone (purity ≥ 99.9%), ethanol (purity ≥ 99.5%) were purchased from Sigma-Aldrich (Poznan, Poland) and used as supplied. All solutions were prepared from high purity reagents and distilled water.

### 2.1. Oxide Film Preparation on Ti6Al4V ELI Transpedicular Screws

Prior to the anodizing process, Ti6Al4V ELI screws were sequentially cleaned for 10 min in acetone, ethanol, and deionized water using an ultrasonic bath and dried in nitrogen.

The anodic oxidation treatment was performed by using the potentiostat/galvanostat AUTOLAB model PGSTAT-302N from AutoLab (EcoChemie, Utrecht, The Netherlands) connected to a two-electrode system, with a cylindrical platinum as cathode and the Ti6Al4V ELI screws as anode. The formation process of compact and nanotubular oxide layer consists of two stages: the first potentiodynamic and the second potentiostatic according to parameters presented in Table 1.

The compact oxide layer was formed on the Ti6Al4V ELI screw during anodizing in 1 M H_3_PO_4_ solution at 20 V with 0.5 V/s scan rate for 20 min.

The nanotubular oxide layer formation: at the beginning, the screws were polarized up to the set potential (22 V) with a 0.5 V/s scan rate and then kept at the set potential (22 V) for further anodizing time (20 min) in ethylene glycol aqueous solution (99%) with 0.6 wt.%. NH_4_F.

At the end of each process, the samples were washed in deionized water.

### 2.2. Implantation of Ti6Al4V ELI Transpedicular Screws

The Ti6Al4V ELI transpedicular screws were used to study delamination and damage of passivate, compact, and nanotubular oxide layers upon implantation into the Th8 vertebra from fully grown pigs. Fresh spines were harvested, cleaned for soft tissues, and stored frozen at a temperature of −20 °C. Prior to the testing day, spines were defrosted at room temperature for 24 h. Th8 vertebrae were chosen to be examined due to the commonly used stabilization in thoracolumbar vertebra and lower holding power of transpedicular screws in thoracic vertebrae compared to lumbar one [24]. According to the Magerl technique, in each vertebrae convergent 40-mm pilot holes for transpedicular screws were made by means of a 3-mm drill [25]. Afterwards, transpedicular solid fully threaded screws were inserted through a vertebral body. The Ti6Al4V ELI screws were then removed and cleaned and further used to examine degradation and chemical stability of an oxide layer after implantation.

### 2.3. Degradation and Chemical Stability Analysis

The sample surface was studied using field emission scanning electron microscopy (FE-SEM, JEOL JSM-7600F, Tokyo, Japan) and energy-dispersive X-ray spectroscopy (EDS, Oxford INCA).

Chemical stability of a transpedicular screw covered by varying types of TiO_2_ oxide layer before and after implantation were characterized by open circuit potential (OCP) and electrochemical impedance spectroscopy (EIS) measurements. OCP measurements were recorded for 1200 s. EIS spectra were measured over a frequency range of 10^5^–0.1 Hz with an acquisition of 10 points per decade and with a signal amplitude of 10 mV. In order to select the equivalent circuit the Nova 2.1.4 software (EcoChemie, Utrecht, The Netherlands) was used.

The AutoLab (EcoChemie, Utrecht, The Netherlands) PGSTAT-302N potentiostat/galvanostat was used to perform these experiments. All measurements were performed in the standard three-electrode configuration with Ti6Al4V ELI transpedicular screw as the working electrode, the standard silver chloride electrode (E_Ag/AgCl_ = 0.222 V vs. Standard Hydrogen Electrode, SHE) as the reference electrode, and a platinum foil as the auxiliary electrode in 0.9% NaCl solution and 0.01 M PBS solution (pH 7.4) at 25 ± 2 °C.

## 3. Results and Discussion

### 3.1. Anodizing of Ti6Al4V ELI Transpedicular Screws

**Figure 1:**
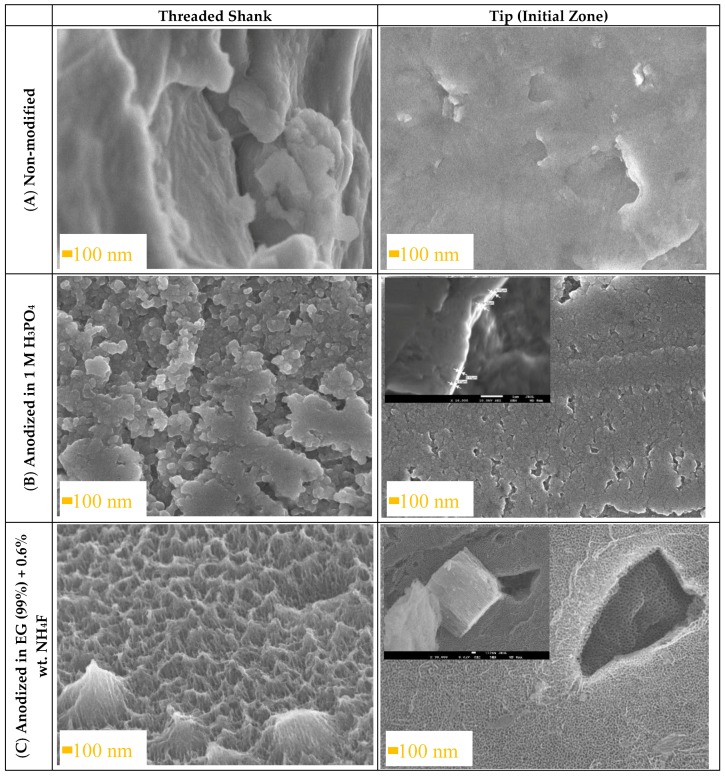
SEM images showing the threaded shrank and tip of Ti6Al4V ELI transpedicular screws: (**A**) non-modified, (**B**) anodized in 1 M H_3_PO_4_ and (**C**) anodized in ethylene glycol (99%) + 0.6 wt.%. NH_4_F before implantation.

Surface modifications are known to improve surface morphology and chemistry of titanium alloys. Anodic layers were formed on Ti6Al4V ELI transpedicular screws in an anodizing process according to the parameters shown in Table 1. The microscopic analysis of the Ti6Al4V ELI screw was performed in a cross section and top-view on tip (initial zone), and on the threaded shank of the screw. The FE-SEM micrographs of the bare Ti6Al4V ELI transpedicular screws, Ti6Al4V ELI transpedicular screws anodized in inorganic electrolyte, and Ti6Al4V ELI transpedicular screws anodized in organic electrolyte with fluoride addition were shown in Figure 1.

Morphology of the substrate surface of Ti6Al4V ELI transpedicular screw shown in Figure 1A is very smooth when compared with an anodized (Figure 1B,C) one. The Ti6Al4V ELI screw anodized in 1 M H_3_PO_4_ shown in Figure 1B presents thick continuous oxide of TiO_2_, with thickness of 100 ± 10 nm, similar to the research carried out by Kumari and Krishna [26]. Formation of compact TiO_2_ was performed in phosphoric acid due to the potential presence of phosphate inclusion possible to obtain at higher anodizing potentials, which promote its resistance to cell adhesion [27,28].

As can be clearly noted in Figure 1C, the Ti6Al4V ELI screw anodized in EG (99%) + 0.6 wt.%. NH_4_F results in the growth of the TiO_2_ nanotubes perpendicular to the surface of the anodized sample. FE-SEM images show arrays of opened from the top, closed at the bottom, and vertically oriented nanotubes. The nanotubular layer is entirely formed of screw surface covered uniformly both the threaded shrank and tip of screw. The mean outer diameter of nanotubes in α-phase was 35 ± 5 nm, while in the β-phase was 50 ± 5 nm. The differences in diameter of TNT on α and β-phases result in favoring adsorption of anions and dissolving well in fluoride ions of vanadium [9]. The layer has average total thickness of length 1500 ± 100 nm. The use of high concentration of ethylene glycol and low pH of this electrolyte by the addition of a high concentration of fluoride allowed to form homogenous TNT in α and β-phases, which has not been obtained yet [14,15], or the layer was cracked [28].

The obtained morphology of nanotubular layer is compatible with reports on cell adhesion on TiO_2_ nanotubes: a small TNT diameter (~30 nm) stimulated the highest degree of osteoblast adhesion [29], diameters of 15–20 nm increased cell adhesion, proliferation as well as alkaline phosphatase (ALP) activity and bone matrix deposition [30]; higher spreading of cytoskeletal actin was found on the surface with a small nanotube diameter (30 nm) [31]. To our knowledge, the influence of titania nanotubes length on cell adhesion has not yet been reported. However, we assume that the length of applied nanotubular substrates does not influence the outcome of the microbial tests because the cells are in contact with the open tube ends. Furthermore, it has been theoretically calculated that the ideal implant surface features at the micro-scale should be 1500 nm long [32,33]. Therefore, the parameter of anodizing process in EG with F^-^ addition was determined using the elaborated mathematical model [34] to ensure the length of TNT being 1500 nm.

Results of the EDS analysis shown in Table 2 confirm the presence of titanium, aluminium, vanadium, and oxygen in a layer formed on the Ti6Al4V ELI transpedicular screw. As it can be seen, the presence of oxygen was observed only for an anodized layer, and the weight percentage correlates with the length of the oxide layer, i.e., is higher for nanotubular layer (~1500 nm) than compact layer (~200 nm). As a result, the weight percentage of titanium in these samples decreased to the stoichiometric, nearly perfect TiO_2_ for nanotubular oxide. In the case of implants, the stoichiometric defects and low stability of this film can lead to their delamination and loosening [19,22]. For a nanotubular oxide layer formed on the Ti6Al4V ELI screw, the division into an α-phase rich in aluminum and β-phase rich in vanadium was observed. Despite the presence of toxic vanadium in the β-phase of Ti6Al4V ELI, the anodizing process caused its oxidation reducing toxic effect from a metal substrate [2,35,36].

The oxide layer prepared on Ti6Al4V ELI transpedicular screws are hereinafter designated as: “Ti6Al4V” for bare transpedicular screw, “Compact Ti6Al4V” for transpedicular screw anodized in 1 M H_3_PO_4_ resulting in compact oxide formation and “Nanotubular Ti6Al4V” for transpedicular screw anodized in ethylene glycol (99%) with 0.6 wt.% NH_4_F addition resulting in nanotubular layer formation.

### 3.2. Optical Assessment of Oxide Layer Degradation after Implantation

Previous attempts to elaborate the mechanical degradation of TNT during implantation were carried out using flat foil [9,14,15,16,17], on Ti, Ti6Al4V, and Ti6Al7Nb screws [19,20,21,22,23] using a nanoindenter test [37], wear test [6,36], or practical implantation of the screws to the bone. The implantation was carried out by inserting a screw into a bone and removing it, which caused double abrasion of the nanotube layer, and prevented TNT characterization inside the bone immediately after implantation. Therefore, the implantation using the Magerl technique was carried out in the pig’s Th8 vertebra and removed not by unscrewing but by drilling through the bone.

**Figure 2:**
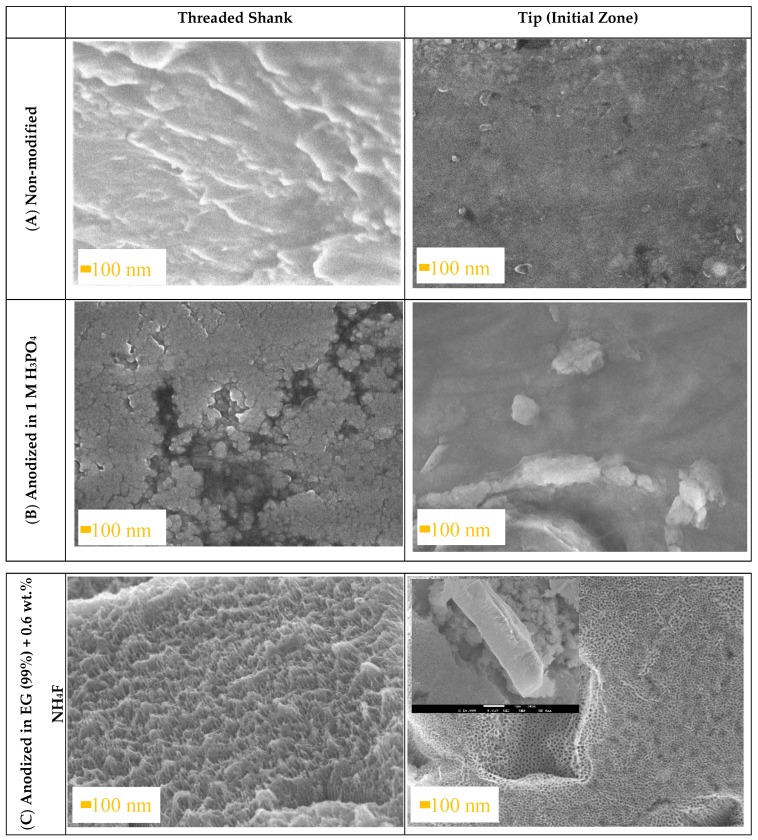
SEM images showing the threaded shrank and tip of Ti6Al4V ELI transpedicular screws: (**A**) non-modified, (**B**) anodized in 1 M H_3_PO_4_ and (**C**) anodized in ethylene glycol (99%) + 0.6% wt. NH_4_F after implantation into pig’s Th8 vertebra by the Magerl technique.

The FE-SEM images of a Ti6Al4V ELI transpedicular screw modified by varying types of oxide layers after the implantation process are shown in Figure 2. As it can be seen, the Ti6Al4V (Figure 2A) and compact Ti6Al4V (Figure 2B) have been abraded and smoothed out after implantation. The evolution of length of a compact layer was impossible due to its non-homogeneity and low height. After implantation, round scratches were observed on the tip of the compact Ti6Al4V screw.

Degradation of morphology of the nanotubular Ti6Al4V (Figure 2C) was not observed after implantation. FE-SEM images show arrays of opened from the top, closed at the bottom, and vertically oriented nanotubes. The microscopic analysis of Ti6Al4V ELI surface does not show any signs of delamination or macroscopic damage of nanotubular Ti6Al4V. Despite that, the length of nanotubular layer was insignificantly decreased from 1500 ± 100 nm to 1300 ± 100 nm and the “hills” created at the top of the surface caused by its high length have been smoothed. This phenomenon was not observed in previous attempts [19,38] due to lower thickness of the nanotubular layer. This is favorable due to the osseointegration process because the surface roughness of nanotubular Ti6Al4V ELI are able to slightly increase after implantation [1].

### 3.3. Electrochemical Examination of Ti6Al4V ELI Transpedicular Screw after Implantation

Metals from orthopaedic implants are released into the surrounding tissue by various mechanisms, including corrosion, wear, and mechanically accelerated electrochemical processes such as stress corrosion, corrosion fatigue, and fretting corrosion [39,40]. The electrochemical tests like open circuit potential measurements and electrochemical impedance spectroscopy help to reveal the microrupture, which is not seen on FE-SEM images. Two significant factors concerning corrosion potential and corrosion resistance are measured by OCP and EIS methods.

#### 3.3.1. Open Circuit Potential Measurements

**Figure 3:**
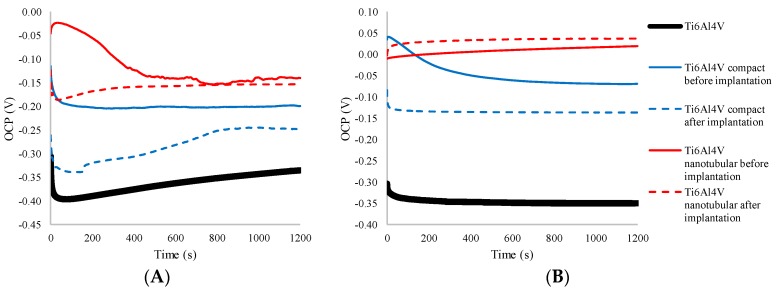
Open circuit potential plots of non-modified (Ti6Al4V), anodized in 1 M H_3_PO_4_ (Ti6Al4V compact) and anodized in ethylene glycol (99%) + 0.6 wt. % NH_4_F (Ti6Al4V nanotubular) before and after implantation into pig’s Th8 vertebra by the Magerl technique measured in 0.9% NaCl (**A**) and 0.01 M PBS (**B**) during 1200 s.

The evaluation of open circuit potential of Ti6Al4V ELI transpedicular screw and covered by compact and nanotubular layer Ti6Al4V ELI transpedicular screw was carried out in 1200 s both in the 0.9% NaCl chloride environment, as well as in 0.01 M PBS solution, which can be used to simulate the body environment due to its high chloride concentration [41]. Typical OCP curves for the analyzed surface on Ti6Al4V ELI screw were presented in Figure 3 and its statistical analysis was presented in Table 3. OCP curves presented in Figure 3A revealed that corrosion potential for compact and nanotubular oxide layer were stabilized in contrast to bare Ti6Al4V ELI. This behavior strongly indicates possible formation of a passive film on bare Ti6Al4V with time.

Bare Ti6Al4V ELI transpedicular screw exhibits a pronounced increasing from −0.40 V/Ag/AgCl towards more positive values until it does not reach a constant value of OCP −0.337 V/Ag/AgCl in 1200 s in 0.9% NaCl. This phenomenon can be explained, as reported by Atmani et al. [7], by the formation of a protective passive oxide film on the surface of the Ti6Al4V alloy. The as-anodized corresponding OCP plot behaves nearly the same as the substrate.

The anodizing process significantly changes the corrosion potential of Ti6Al4V ELI from −0.337 V (bare) to −0.193 V for compact Ti6Al4V and to −0.129 V for nanotubular Ti6Al4V measured in 0.9% NaCl. This initial increase seems to be related to the formation and thickening of the oxide film on the metallic surface, improving its corrosion protection ability [15]. It should be mentioned that a more positive corrosion potential results in higher corrosion resistance of the coating, confirming the possibility to reduce a toxic effect from a metal substrate by better corrosion protection of anodic layers [42]. On both forms, the oxide layer indicates higher corrosion potential compared to bare Ti6Al4V ELI, which confirms its better protection properties against corrosion fatigue, fretting corrosion, and stress corrosion. The most stable potential indicated the compact Ti6Al4V before implantation, confirming the homogeneity of this layer. Oscillations seen in the course of OCP measurements of nanotubular Ti6Al4V before implantation are the results of wettability of TNT [8,9] in such a long structure. Despite this, stability in the corrosion potentials of a nanotubular Ti6Al4V ELI screw from 400 s of measurement indicates that this layer became thermodynamically stable with time.

On both the compact and nanotubular layers, the corrosion potential decreased after implantation, what confirms a small microrupture of these layers. The decrease of OCP for compact Ti6Al4V was 50 mV and for nanotubular Ti6Al4V was 25 mV after implantation, compared to its original values. It confirms better mechanical stability of the nanotubular layer, and lower degradation of this layer during the implantation process [36]. For compact Ti6Al4V, the stabilizing time of OCP was longer than nanotubular, i.e., 900 s, and the amplitude of corrosion potential was much greater, showing greater damage of this layer.

The above-mentioned correlations were also observed in OCP curves measured in PBS solution, presented in Figure 3B. The higher values of OCP recorded for each type of sample are the result of a higher pH of PBS solution (7.4 compared to 5.4 for 0.9% NaCl) and the presence of phosphate ions. Metals typically develop a passivation layer in moderately alkaline solutions (PBS), which lower the corrosion rate as compared to acidic (NaCl) solutions, which explains the more stable curves recorded for each sample. Cl-concentration strongly affects the corrosion rate inside the tested solutions, whereas higher concentration was found in 0.9% NaCl solution. The presence of phosphate ions in electrolytes increases the inhibition effect and corrosion resistance of analyzed transpedicular screws.

#### 3.3.2. Electrochemical Impedance Spectroscopy Measurement

**Figure 4:**
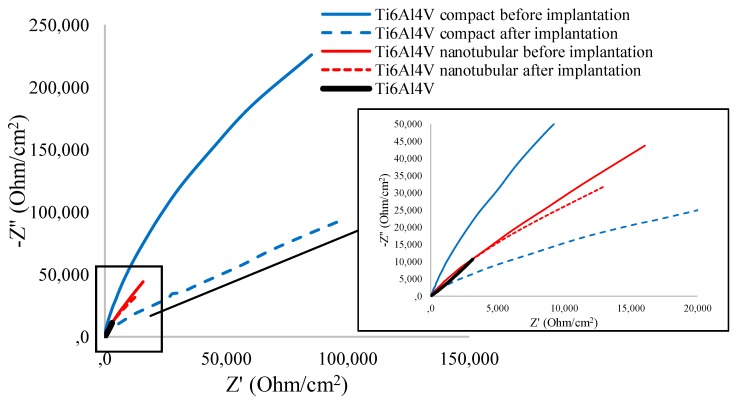
Nyquist diagrams for the Ti6Al4V ELI transpedicular screw: non-modified (Ti6Al4V), anodized in 1 M H_3_PO_4_ (Ti6Al4V compact) and anodized in ethylene glycol (99%) + 0.6 wt.% NH_4_F (Ti6Al4V nanotubular) before and after implantation into pig’s Th8 vertebra by the Magerl technique measured in 0.9% NaCl in frequency range 0.1–10^5^ Hz.

**Figure 5:**
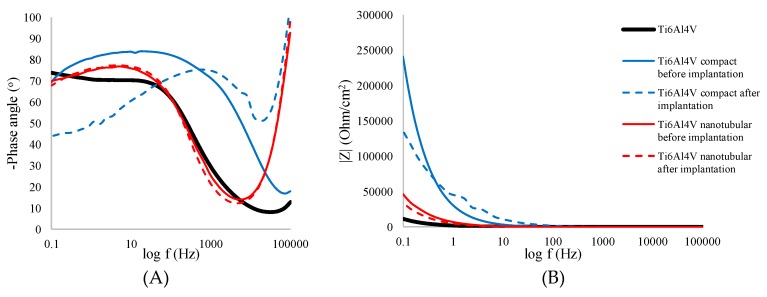

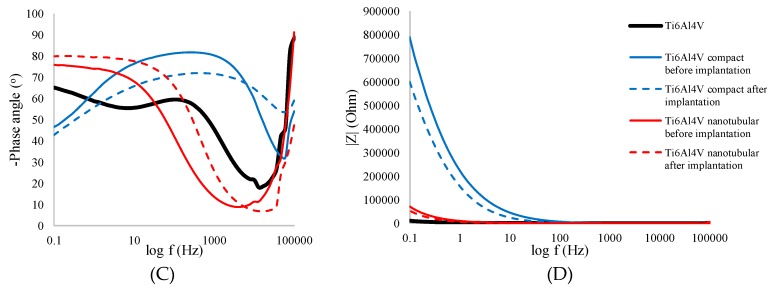
Bode plots for non-modified (Ti6Al4V), anodized in 1 M H_3_PO_4_ (Ti6Al4V compact) and anodized in ethylene glycol (99%) + 0.6 wt.% NH_4_F (Ti6Al4V nanotubular) before and after implantation into pig’s Th8 vertebra by the Magerl technique measured in 0.9% NaCl (**A**,**B**) and in 0.01 M PBS (**C**,**D**) in a frequency range of 0.1–10^5^ Hz.

EIS tests were carried out to further study the electrochemical characteristics of transpedicular screw and its degradation during implantation. The EIS plots and simulated curves for the bare, compact, and nanotubular Ti6Al4V ELI screws are shown in Nyquist representation in Figure 4 and in Bode representation in Figure 5. The summary of impedimetric parameters of bare, compact, and nanotubular Ti6Al4V are shown in Table 4.

The Nyquist curves shown in Figure 4 present an incomplete semicircle in the entire frequency range, a typical impedance response of thin oxide layers. The capacitance loop diameter (Figure 4) of the bare Ti6Al4V is substantially greater than anodized Ti6Al4V transpedicular screws. Furthermore, the compact Ti6Al4V exhibits more capacitive behavior than the nanotubular Ti6Al4V layer. The further depressed capacitive semicircles are associated with the charge transfer process in the electrode/electrolyte interface.

The decrease of the semi-circular diameter of recorded for compact and nanotubular Ti6Al4V screws after implantation confirms increased electrolytic resistance and deterioration of corrosion resistance. The implantation process caused the significant change in active and passive resistance of the compact layer, the real impedance (ReZ) decreased more than twice from 85,318.6 to 194,309.6 Ω/cm^2^, while the imaginary impedance (ImZ) decreased from 225,726.6 to 183,136.7 Ω/cm^2^. The increase of active resistance confirms the microrupture in compact oxide layer after implantation.

Both ReZ and ImZ decreased after implantation of nanotubular Ti6Al4V; however, these changes are smaller when compared to a compact one. It should be noted that compact and nanotubular layers significantly increase the semicircle radius (even after the implantation process), indicating an enhanced stability of passive film for corrosion resistance [21].

Bode representation of impedance spectra of bare, compact, and nanotubular Ti6Al4V measured in 0.9% NaCl, shown in Figure 5a,b, confirms the increase of impedance of compact and nanotubular layer compared to bare Ti6Al4V. The implantation process caused a slight decrease of impedance which does not exceed 10% for compact and 20% for the oxide layer.

The changes in morphology and heterogeneity of compact and nanotubular layer are clearly seen in phase angle (Zphase) changes (Figure 5A). The nanotubular Ti6Al4V before and after implantation has a very similar course, and the Zphase values do not change significantly, from −69.81 to −67.79°. The one time constant confirms the presence and heterogeneity of nanotubular oxide layer. Compact oxide Ti6Al4V dramatically changes after the implantation process, the one-time constant change from 100 to 1000 Hz, and the Zphase decreased from −69.29 to −43.30°.

Bode representation of impedance spectra of bare, compact, and nanotubular Ti6Al4V measured in 0.01 M PBS, shown in Figure 5C,D, indicate the same correlation as described above. Implantation causes a decrease in phase angle value and impedance modulus, both for compact and nanotubular Ti6Al4V. Higher phase angle values recorded for nanotubular Ti6Al4V are the result of a higher affinity to phosphate absorbing by titanium dioxide nanotubes [28,42,43]. Therefore, as can be seen in Table 4, the larger capacitive semicircle diameters and the increased phase angle all imply that a more protective passive layer was obtained on the nanotubular Ti6Al4V.

**Figure 6:**
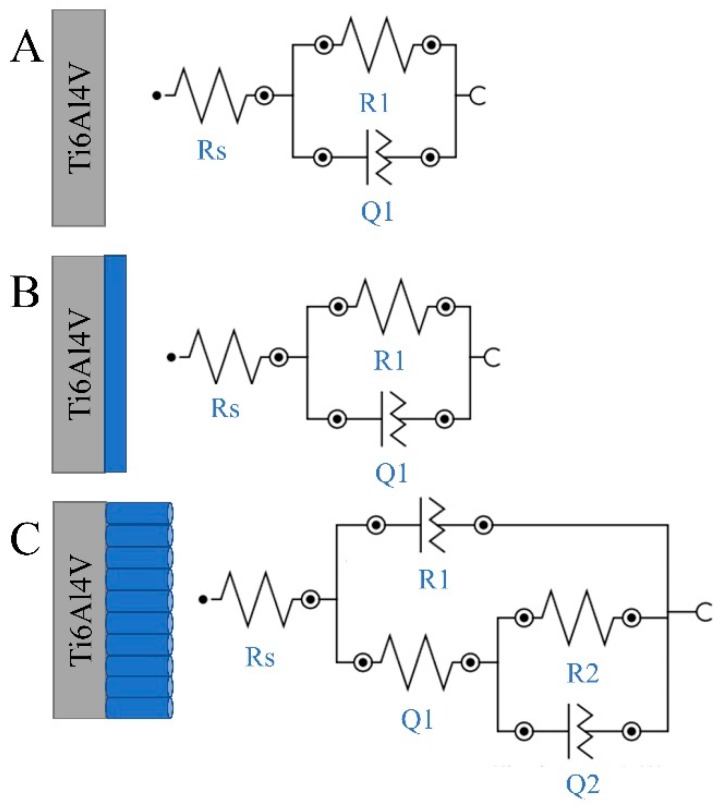
Equivalent circuits used to model the impedance spectra of the bare (**A**), compact (**B**), and nanotubular (**C**) samples of a Ti6Al4V transpedicular screw.

The specific polarization resistance was calculated following the fitting of the impedance experimental results considering a solid electrode (bare Ti6Al4V, anodized Ti6Al4V) in contact with 0.9% NaCl electrolyte solution. The results of impedance investigations were fitted to the equivalent circuits (Figure 6), which represent a single-layer model—bare and compact Ti6Al4V ELI—and a bi-layer model—nanotubular Ti6Al4V and the interior of the nanotubes. Identical equivalent circuits have already been used in literature for compact and nanotubular oxide layer [8,44]. The Rs element represents the uncompensated solution resistance relaxing at high frequencies. Due to the surface heterogeneities of the treated samples, as shown in Figure 1 and Figure 2, a constant phase element Q is used to fit the data instead of a pure capacitor C [44]. Parallel combination R1Q1 represents the resistance and constant phase element with the capacitance C1 of the porous TiO_2_. The next parallel combination R2Q2 determining TiO_2_ nanotubes layer leads to a depressed semicircle in the corresponding Nyquist impedance plot (Figure 4) [30].

The parameters determined by fitting of the experimental EIS data are summarized in Table 5. The equivalent circuits allow a good agreement between the experimental data and the simulated impedance plots for comparative estimation of specific components of the studied surfaces. The resistance, Rs, decreased in the following order: bare Ti6Al4V ELI, compact Ti6Al4V ELI, and nanotubular Ti6Al4V ELI before implantation, confirming the differences in electrical conductivity of the analyzed samples, and its improving by anodization treatment.

According to the data presented in Table 5, a nanotubular Ti6Al4V layer consists of a thin inner compact and a barrier titanium oxide layer followed by an outer nanotubular layer. This result confirms the data obtained in previous studies [44]. With regard to the present tubular structure, the corrosion resistance of the nanotubular Ti6Al4V is lower than that of the other specimens (Table 3). The value of N corresponds to the linear slope modulus of Bode plot (Figure 5B). It is well known that, when N is near 1, the surface is uniform and smooth. On the other hand, lower values (in our case N = 0.92 in the compact Ti6Al4V specimen, and *n* = 0.86 in the nanotubular Ti6Al4V specimen) show deviation from ideal capacitive behavior (which has been attributed to the inhomogeneity of the surface) and deterioration of corrosion resistance [44]. Due to this result, the highest polarization resistance (extrapolation of Z modulus at low frequency) is obtained for a nanotubular interface formed on the Ti6Al4V transpedicular screw (Figure 5B). Furthermore, this result confirms the FE-SEM micrograph and shows that formation of titania nanotubes on the Ti6Al4V transpedicular screw causes the increase of corrosion resistance.

## 4. Conclusions

The Ti6Al4V ELI transpedicular screws were anodized resulting in a compact oxide layer (height: 100 ± 10 nm) and nanotubular oxide layer (outer diameter of nanotubes in the α-phase was 35 ± 5 nm, while, in the β-phase, it was 50 ± 5 nm, total thickness of length 1500 ± 100 nm) formation. The anodic layers were homogenously formed on tip and on the threaded shank of the screw, which was confirmed by scanning electron microscopy pictures. According to the literature, obtained morphology was favorable to enhanced osteointegration and antibacterial action.

The implantation process of a transpedicular screw included single screwing through the pig’s Th8 vertebra, making it possible to characterize the analyzed layer immediately after implanting. The FE-SEM analysis showed the round scratches on the tip of compact Ti6Al4V screw after implantation. The nanotubular layer was smoothed after implantation, and free of “hills” characteristic to high-length nanotubes.

More accurate analysis of degradation of anodic layers was performed by electrochemical characteristics in sodium chloride and phosphate buffered solutions. The anodic layer was characterized by higher open circuit potential values and more capacitive behavior compared to bare Ti6Al4V ELI, proving its better corrosion resistance and protection. Implantation caused the corrosion potential decreased after implantation, confirming a small microrupture of the anodic layer. The decrease of OCP for compact Ti6Al4V was 50 mV and, for nanotubular Ti6Al4V, was 25 mV after implantation, compared to its original values. It confirms better mechanical stability of a nanotubular layer, and lower degradation of this layer during the implantation process. For compact Ti6Al4V, the stabilizing time of OCP was longer than nanotubular, i.e., 900 s, and the amplitude of corrosion potential was much greater, showing greater damage of this layer. These results were confirmed by a fitted equivalent circuit.

In conclusion, the obtained results indicate degradation of the anodic layer, both compact and nanotubular after the implantation process. Much greater degradation and microruptures were observed in a compact oxide layer, making the nanotubular oxide layer clinically relevant. These results are in contrast to previous studies, strongly showing the significance of toxicological studies of layer splinters.

## Figures and Tables

**Table 1:** Summary of parameters used during the anodic oxidation process of the Ti6Al4V ELI transpedicular screw.

Samples	Solution	Voltage [V]	Time [min]
Ti6Al4V	-	-	-
Compact Ti6Al4V	1 M H_3_PO_4_	20	20
Nanotubular Ti6Al4V	EG + 0.6 wt. % NH_4_F + 1 wt.% H_2_O	22	20

**Table 2:** Chemical composition of the Ti6Al4V ELI transpedicular screws: (**A**) non-modified, (**B**) anodized in 1 M H_3_PO_4_ and (**C**) anodized in ethylene glycol (99%) + 0.6% wt. NH_4_F.

Ti6Al4V ELI Transpedicular Screws		Ti [wt. %]	Al [wt. %]	V [wt. %]	O [wt. %]
(A) Non-modified		91.15	5.96	3.53	---
(B) Anodized in 1 M H_3_PO_4_		72.6	4.56	3.6	19.19
(C) Anodized in EG (99%) + 0.6 wt. % NH_4_F	α-phase	63.79	2.34	---	33.96
	β-phase	52.7	1.24	28.98	17.13

**Table 3:** Open circuit potential measurements with statistical analysis (n = 5).

	OCP in NaCl [V]	RSD [%]	OCP in PBS [V]	RSD [%]
**Ti6Al4V**	−0.337	4.25	−0.319	3.29
**Compact Ti6Al4V before implantation**	−0.193	4.74	−0.116	4.55
**Compact Ti6Al4V after implantation**	−0.242	3.47	−0.068	5.83
**Nanotubular Ti6Al4V before implantation**	−0.129	7.82	0.021	6.37
**Nanotubular Ti6Al4V after implantation**	−0.154	5.11	0.037	5.23

Ti6Al4V—bare Ti6Al4V ELI transpedicular screws; OCP—Open Circuit Potential; RSD—Relative Standard Deviation; n—Number of samples

**Table 4:** Impedimetric parameter of Ti6Al4V measured in 0.9% NaCl with statistical analysis (n = 5).

	|Z| [Ω/cm^2^]	RSD [%]	-Zphase [^o^]	RSD [%]	ReZ [Ω/cm^2^]	RSD [%]	-ImZ [Ω/cm^2^]	RSD [%]
**Ti6Al4V**	11358	5.45	73.39	1.65	3078	2.04	10594	4.65
**Before implantation**								
**Compact Ti6Al4V**	244399	7.68	69.29	1.80	85319	4.61	225727	7.72
**Nanotubular Ti6Al4V**	44261	6.43	69.81	2.05	16072	9.76	43697	6.39
**After implantation**								
**Compact Ti6Al4V**	264887	8.53	43.30	2.98	194309	5.18	183137	7.36
**Nanotubular Ti6Al4V**	37563	7.71	67.79	1.93	12994	9.13	31831	8.58

Ti6Al4V—bare Ti6Al4V ELI transpedicular screws

**Table 5:** Value of circuit equivalent elements (abbreviation according to models in Figure 6) for non-modified (Ti6Al4V), anodized in 1 M H_3_PO_4_ (Ti6Al4V compact) and anodized in ethylene glycol (99%) + 0.6 wt. % NH_4_F (Ti6Al4V nanotubular) before and after implantation into pig’s Th8 vertebra by the Magerl technique.

Element		Ti6Al4V	Compact Ti6Al4V before Implantation	Compact Ti6Al4V after Implantation	Nanotubular Ti6Al4V before Implantation	Nanotubular Ti6Al4V after Implantation
Rs [Ω]		9.36	7.29	7.30	1.59	2.05
R1 [Ω]		1.00 × 10^6^	1.00 × 10^6^	9.00 × 10^5^	1.97 × 10^5^	1.20 × 10^4^
Q1	C1 [F]	1.30 × 10^−4^	6.10 × 10^−6^	8.47 × 10^−6^	2.84 × 10^−5^	3.89 × 10^−5^
	N1	0.79	0.92	0.55	0.86	0.87
τ_1_ = R1·C1 [s]		130	6.1	7.62	5.59	0.47
R2 [Ω]					2.64 × 10^5^	0.69 × 10^5^
Q2	C2 [F]				1.08 × 10^−5^	2.19 × 10^−5^
	N2				0.98	0.99
τ_1_ = R2·C2 [s]					2.85	1.51
	χ²	0.016	0.0301	0.0320	0.0046	0.009
